# The effectiveness of styrene-maleic acid (SMA) copolymers for solubilisation of integral membrane proteins from SMA-accessible and SMA-resistant membranes

**DOI:** 10.1016/j.bbamem.2017.07.011

**Published:** 2017-10

**Authors:** David J.K. Swainsbury, Stefan Scheidelaar, Nicholas Foster, Rienk van Grondelle, J. Antoinette Killian, Michael R. Jones

**Affiliations:** a School of Biochemistry, University of Bristol, Biomedical Sciences Building, University Walk, Bristol BS8 1TD, United Kingdom; b Membrane Biochemistry & Biophysics, Utrecht University, Bijvoet Center for Biomolecular Research, Utrecht, The Netherlands; c Division of Physics and Astronomy, VU University Amsterdam, De Boelelaan 1081, Amsterdam 1081 HV, The Netherlands

**Keywords:** Styrene—maleic acid, Nanodisc, Membrane protein, Detergent, Reaction centre, Light harvesting

## Abstract

Solubilisation of biological lipid bilayer membranes for analysis of their protein complement has traditionally been carried out using detergents, but there is increasing interest in the use of amphiphilic copolymers such as styrene maleic acid (SMA) for the solubilisation, purification and characterisation of integral membrane proteins in the form of protein/lipid nanodiscs. Here we survey the effectiveness of various commercially-available formulations of the SMA copolymer in solubilising *Rhodobacter sphaeroides* reaction centres (RCs) from photosynthetic membranes. We find that formulations of SMA with a 2:1 or 3:1 ratio of styrene to maleic acid are almost as effective as detergent in solubilising RCs, with the best solubilisation by short chain variants (< 30 kDa weight average molecular weight). The effectiveness of 10 kDa 2:1 and 3:1 formulations of SMA to solubilise RCs gradually declined when genetically-encoded coiled-coil bundles were used to artificially tether normally monomeric RCs into dimeric, trimeric and tetrameric multimers. The ability of SMA to solubilise reaction centre-light harvesting 1 (RC-LH1) complexes from densely packed and highly ordered photosynthetic membranes was uniformly low, but could be increased through a variety of treatments to increase the lipid:protein ratio. However, proteins isolated from such membranes comprised clusters of complexes in small membrane patches rather than individual proteins. We conclude that short-chain 2:1 and 3:1 formulations of SMA are the most effective in solubilising integral membrane proteins, but that solubilisation efficiencies are strongly influenced by the size of the target protein and the density of packing of proteins in the membrane.

## Highlights

• Styrene maleic acid (SMA) solubilises reaction centres from antenna-free membranes.
• SMA with 2:1 or 3:1 ratios of styrene to maleic acid are the most effective.
• SMA is poor at solubilising densely packed and highly ordered membranes.
• A variety of lipid dilution treatments improve solubilisation efficiencies.
• Solubilised material from SMA-recalcitrant membranes comprises small membrane patches.

## Graphical abstract

**Figure:**
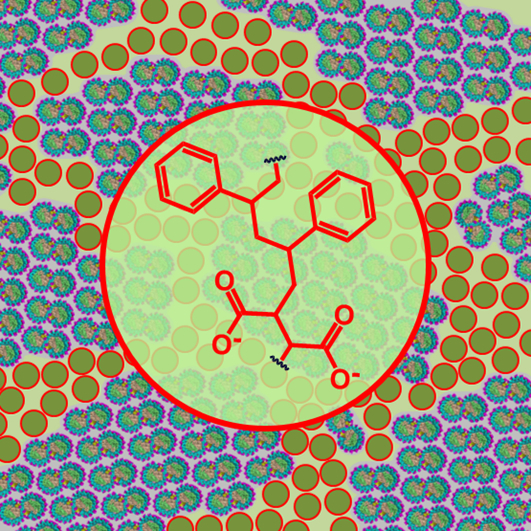

## 1 Introduction

Styrene—maleic acid (SMA) is a copolymer of styrene and maleic acid moieties that shows great promise as an alternative to detergents for the solubilisation, purification and characterisation of integral membrane proteins [1], [2], [3]. Unlike detergents, which tend to strip away most or all of the lipids in the immediate environment of a membrane protein, SMA extracts proteins in the form of a lipid/protein nanodisc [4]. These typically range in size from 10 to 15 nm, and estimates of the number of lipids they contain have ranged from 11 to 150 (see [2] for a review). A number of recent studies have shown that SMA can be used to produce highly pure preparations of integral membrane proteins from a variety of bacterial and eukaryotic sources [5], [6], [7], [8], [9], [10], [11], [12], [13], [14]. Modification with a His-tag greatly assists this process by providing a means to separate nanodiscs containing the target protein from the heterogeneous population produced from a solubilised membrane. SMA has also been used to prepare multicomponent membrane protein complexes that are not stable in detergent [15], [16], and to investigate transient associations of membrane proteins during the formation of metabolons in plant cell membranes [17].

In recent reports, Lee and co-workers have commented that there may be size limits on proteins that can be solubilised using SMA [3], [18]. On the basis of purification of more than thirty membrane proteins, they suggested that proteins with > 36 to 40 transmembrane α-helices may not be extractable using SMA, the constraint being the maximum diameter (~ 15 nm) of the SMA/lipid discs that can be formed. This may not be a fixed limit however, as particle sizes of 18 nm and 24 nm were reported for nanodiscs containing various proteins from *Staphylococcus aureus* [7], nanodiscs of 25 nm were reported in studies of metabolon complexes from *Sorghum bicolor* [17] and a wide range of nanodisc sizes were also reported for solubilised mitochondrial membranes [19]. In studies of SMA-solubilisation of 1,2-dimyristoyl-sn-glycero-3-phosphocholine (DMPC) bilayers it has reported that nanodisc size can depend on the molar ratio of SMA to lipid, with discs of ~ 13 nm diameter being seen at a SMA:DMPC ratio of 0.5 but ~ 29 nm discs being seen at a ratio of 0.17 [20]. Moreover, Craig and co-workers have recently reported empty discs of up to 32 nm using a RAFT polymerised SMA with a different polymer structure to the commercially available SMAs employed here and in other studies [21].

In previous work [8] we have shown that SMA can be used to extract and purify the reaction centre (RC) pigment-protein from membranes from a strain of the purple photosynthetic bacterium *Rhodobacter* (*R.) sphaeroides* lacking both types of light harvesting “antenna” complex [22] (see below). This RC complex comprises three polypeptides with a combined total of eleven membrane-spanning α-helices and has a mass of ~ 104 kDa [23], [24], [25], [26]. Use of this antenna-deficient strain of *R. sphaeroides* enabled comparison of the spectroscopic properties of SMA-purified RCs with those of RCs in intact bacterial membranes and purified in detergent [8]. Complete solubilisation of cytoplasmic membranes from this antenna-deficient strain was achieved at room temperature using SMA2000 (Cray Valley, USA), a SMA copolymer with a 2:1 ratio of styrene to maleic acid moieties and a weight average molecular weight of 7.5 kDa [8]. The purified RCs showed a normal pigment absorbance spectrum, a good indication of native structure, and showed functional properties more consistent with RCs in native membranes than RCs in detergent. These findings demonstrated the ability of SMA to preserve aspects of membrane protein function that are altered or lost in detergent [8].

**Fig. 1:**
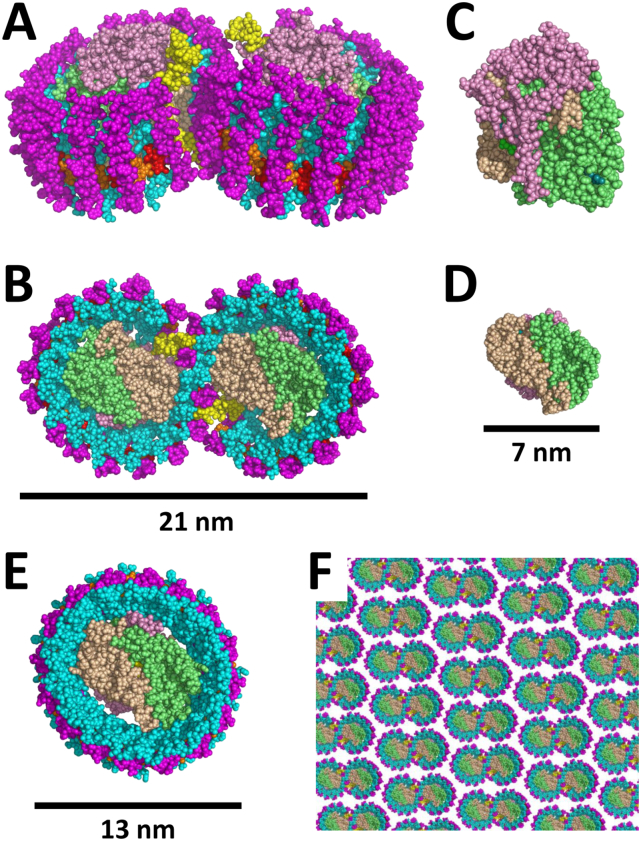
Architectures of membrane proteins used to test SMA solubilisation. (A,B) Space-fill representations of the RC-LH1-X dimer from *R. sphaeroides* at 7.8 Å resolution (PDB entry 4V9G [27]) viewed (A) side-on and (B) perpendicular to the periplasmic side of the membrane. (C,D) Space-fill representations of the *R. sphaeroides* RC at 2.3 Å resolution (PDB entry 3ZUW [71]) viewed (C) side-on and (D) perpendicular to the periplasmic side of the membrane. (E) Space-fill representation of the *Thermochromatium tepidum* RC-LH1 complex at 3.0 Å resolution (PDB entry 3WMM [28]); this complex has the same architecture as the PufX-deficient *R. sphaeroides* RC-LH1 complex with a complete ring of LH1 pigment-protein surrounding the RC. (F) Model of a semicrystalline array of closely-packed dimeric RC-LH1-X complexes based on a Fourier transform of an electron microscopy image of a photosynthetic membrane from an LH2-deficient strain of *R. sphaeroides* (Fig. 3C from Jungas et al. [35]). As the RC-LH1-X dimer is bent along its long axis (see panel A) membranes from such strains have a tubular architecture. In all panels, colour coding is: magenta – LH1 β-polypeptide, cyan – LH1 α-polypeptide, yellow – PufX, red/orange – LH1 bacteriochlorophylls, pink – RC H-polypeptide, lime – RC M-polypeptide, beige – RC L-polypeptide. The models in B, D and E are shown on the same scale.

Here, we explore the extent to which SMA is able to extract larger pigment-protein complexes from antenna-deficient photosynthetic membranes, and from a second type of photosynthetic membrane that displays a high degree of order and dense protein packing. In wild-type strains of *R. sphaeroides* the RC is part of a larger RC-LH1-X complex along with the LH1 light harvesting pigment-protein (Fig. 1A,B). LH1 forms an incomplete hollow cylinder with the RC (Fig. 1C, D) at the centre [27], [28]. Complete closure of the LH1 ring is prevented by the PufX protein (yellow in Fig. 1A, B) [29], [30], [31], [32]. In photosynthetic membranes, these RC-LH1-X complexes associate with each other and with a peripheral LH2 pigment-protein, LH1 and LH2 forming an antenna that feeds the RC with excited state energy to power trans‑membrane electron transfer [33], [34]. Monomers of RC-LH1-X complexes pack together in the membrane with a two-fold symmetry, such that two C-shaped LH1 are arranged as an S-shaped dimer [30] in which two RCs are surrounded and interconnected by a continuous LH1 antenna (Fig. 1B) [27], [31], [35], [36]. The strength of this dimer interaction is rather variable depending on growth conditions, such that even with mild detergents the predominant form of the complex that is isolated from membranes is the RC-LH1-X monomer when cells are grown under dark/semi-aerobic conditions [32], [37], [38]. Removal of the PufX protein by gene deletion results in an exclusively monomeric RC-LH1 complex [30] in which the RC is surrounded by a closed ring of LH1 pigment-protein (Fig. 1E) [29]. A variety of techniques have shown that, in bacterial strains lacking the LH2 antenna, dimeric RC-LH1-X complexes form ordered, protein-rich arrays in the photosynthetic membrane [31], [35], [36], [39], [40]; the packing model in Fig. 1F is based on an electron microscopy image published by Jungas and co-workers [35]. The dimeric RC-LH1-X complex has a bend along its long axis which induces membrane curvature, resulting in ordered and densely-packed membranes with a tubular architecture [31], [36], [39], [40], [41], [42]. Near circular monomeric PufX-deficient RC-LH1 complexes also form extensive, protein-rich arrays that display regular hexagonal packing, forming membranes comprising large vesicles and sheets [31], [43].

Following our recent work on purifying RCs [8] we attempted to use the same formulation of SMA to isolate larger RC-LH1-X and RC-LH1 complexes from photosynthetic membranes containing LH1, or both LH1 and LH2. As documented below, it was found that such membranes were markedly resistant to solubilisation by SMA. This raises questions over whether these RC-LH1-X complexes are too large to be accommodated in a SMA/lipid nanoparticle, whether the densely-packed composition of antenna-containing membranes disfavours permeation by this formulation of SMA, and how the latter may be overcome. To address these questions, we have explored methodologies to vary membrane protein density and tested the consequences for the efficiency of protein extraction from membranes from antenna-containing *R. sphaeroides* strains expressing His-tagged RC-LH1-X complexes. To deconvolute the contributions of size and packing to the SMA-resistance RC-LH1-X membranes, we have also examined SMA-solubilisation from antenna-deficient strains in which the normally monomeric RC is engineered to assemble in dimeric, trimeric or tetrameric forms to produce oligomeric complexes with up to 44 transmembrane α-helices [44]. We also survey the effectiveness of the range of commercially-available SMA copolymers in solubilising RCs and RC-LH1 pigment-proteins from these membranes. We conclude that SMA formulations with a 2:1 or 3:1 ratio of styrene to maleic acid are most effective at solubilising RCs from SMA-amenable membranes, and that solubilisation of SMA-recalcitrant membranes may be improved by increasing their lipid-to-protein ratio to introduce more regions of lipid bilayer.

## 2 Materials and methods

### 2.1 Preparation of SMA copolymers

The eight SMA polymers assessed in this work are detailed in Table 1; molecular weights quoted in the text are weight average molecular weights. Styrene maleic anhydride pellets were mixed at 5% (*w*/*v*) with deionised water in a round bottom flask. Potassium hydroxide was added at a ratio of 0.3, 0.24, 0.2 or 0.14 g KOH per gram SMA for the 1.5:1, 2:1, 3:1 and 4.5:1 polymers, respectively, to give a pH close to 8.0 after hydrolysis. Solutions were heated at 110 °C whilst refluxing with a condenser for 16 h. If solutions of the 80 and 120 kDa polymers had not clarified this period was extended by up to a further 24 h. Clarified solutions were allowed to cool, the pH was adjusted to 8.0 with KOH, and they were stored at 4 °C for up to two weeks until required. The long-chain variants of the 3:1 SMA formulation had reduced solubility compared to the shorter chain SMAs. As well as increasing the time required to convert them to the soluble form this limited the maximum concentrations of stock solution achievable to approximately 3.5–4% (*w*/*v*).

**Table 1:** Properties of SMA preparations used in this work.

Ratio of styrene: maleic acid	Weight average molecular weight (kDa)	Number average molecular weight (kDa)	Commercial name	Supplier
1.47	5	2	Xiran SZ40005	Polyscope
2.00	7.5	3	SMA2000	Cray Valley
2.16	10	2.5	Xiran SZ30010	Polyscope
2.16	30	9	Xiran SZ30030	Polyscope
3.19	10	4	Xiran SZ26030	Polyscope
3.02	80	32	Xiran SZ26080	Polyscope
3.02	120	48	Xiran SZ26120	Polyscope
4.53	11	2.5	Xiran SZ20010	Polyscope

### 2.2 Biological materials

Cells of *R. sphaeroides* strain DD13 [45] were transformed with pRK415-based plasmids expressing native RCs [46], native RC-LH1-X complexes with PufX, [47], modified RC-LH1 complexes without PufX (RC-LH1) [48], and RCs modified with extra-membrane α-helical sequences that form dimeric, trimeric or tetrameric coiled-coil bundles [46]. Complementation was achieved by conjugative transfer from *Escherichia* (*E.*) *coli* strain S17–1 [49] and in all cases the RC component was modified at the C-terminus of the PufM polypeptide with a deca-histidine tag [46]. Bacterial cells were stored as thick suspensions in 70% lysogeny broth/30% glycerol at − 80 °C.

For growth of *R. sphaeroides*, glycerol stocks were used to inoculate a 10 mL starter culture of M22 + minimal medium [49] in a 30 mL universal bottle that was grown for 24 h in the dark at 34 °C and 180 rpm in an orbital incubator. This was then used to inoculate 70 mL of M22 + in a 100 mL conical flask and this culture was grown on for 24 h under the same conditions. This intermediate culture was then used to inoculate 1.5 L of M22 + medium in a 2 L conical flask. This culture was grown for 48 h under the same conditions, and scaled up as necessary. Cells were harvested by centrifugation and cell pellets were stored at − 20 °C until required. For all cells expressing photosynthetic complexes neomycin and tetracycline were added to the media at 100 μg/mL, but cells of strain DD13 were grown in the presence of neomycin only. For growth under high aeration 1 L of M22 + media containing tetracycline and neomycin in a 2 L baffled conical flask was inoculated with an 80 mL intermediate culture, and flasks were incubated overnight in the dark at 34 °C and 250 rpm in an orbital incubator.

For the preparation of photosynthetic membranes, harvested bacterial cells were resuspended in 20 mM Tris (pH 8) containing a few crystals of bovine DNAse I (Sigma). Cells were lysed at 20,000 psi using a cell disruptor (Constant Systems) and debris was removed by centrifugation at 27,000 g at 4 °C for 15 min. The supernatant was overlaid onto a cushion of 60% (*w*/*w*) sucrose in 20 mM Tris (pH 8) and centrifuged at 167,000 g for 2 h at 4 °C. The membrane band was harvested and used fresh or stored at − 20 °C until required.

### 2.3 Solubilisation screen

Solutions of membranes containing RC, RC-LH1-X or RC-LH1 complexes at a concentration of 3 μM were prepared in 20 mM Tris (pH 8) containing 200 mM NaCl. SMA from a 5% (*w*/*v*) stock solution was mixed in a 1:1 (v:v) ratio with 500 μL of membrane solution, an absorbance spectrum recorded between 400 and 1000 nm, and the resulting mix incubated in the dark at room temperature for 1 h. The mixture was then loaded into a 1 mL ultracentrifuge tube and insoluble material was pelleted at 100,000 g for 2 h at 4 °C. The supernatant was carefully removed and an absorbance spectrum recorded. For detergent extraction the SMA was replaced by 2.5% (*w*/*v*) *n*-dodecyl β-D-maltoside (DDM).

As the expression level of artificially multimeric RCs was not uniform the concentrations of membranes containing dimeric, trimeric and tetrameric RCs were adjusted to match the absorbance at 650 nm of membranes containing unmodified monomeric RCs such that the quantity of total membrane material in each membrane suspension was the same before the addition of SMA. Measured absorbance at this wavelength was almost entirely due to light scattering from the membranes as the RC pigments have almost no absorbance at this wavelength. Normalising to an absorbance of 0.6 at 650 nm produced concentrations of ~ 0.5 μM dimeric RC, ~ 1.5 μM trimeric RC and ~ 1 μM tetrameric RC, and these membrane solutions were mixed 1:1 (v:v) with each SMA stock solution.

All absorbance spectra were corrected for light scatter as described previously [8]. Scatter corrected spectra were used to estimate the percentage of complex remaining in the soluble fraction using absorbance values at 803 nm (for RCs and RC multimers) or 875 nm (for RC-LH1-X and RC-LH1 complexes). Protein concentrations were determined using published extinction coefficients for RCs [50] and RC-LH1 complexes [43], [51].

### 2.4 Purification of RCs and RC-LH1 complexes

RC multimers and RC-LH1 complexes solubilised using SMA were purified by nickel affinity chromatography using SMA-free buffers as previously described for the native monomeric RC modified with a deca-histidine tag [8]. The SMA used was Xiran SZ30010 which has a 2:1 styrene:maleic acid formulation and a weight average molecular weight of 10 kDa.

### 2.5 Dynamic light scattering

Purified SMA/lipid/protein nanodiscs were diluted to 2 μM protein concentration in 20 mM Tris (pH 8) containing 200 mM NaCl and passed through a 0.1 μm spin filter. Dynamic light scattering (DLS) was measured at 25 °C in a 200 μL quartz cuvette inserted into a Zetasizer Nano ZS instrument (Malvern). Three data sets consisting of ten repeats of a ten-second measurement were averaged and analysed by volume to estimate the average hydrodynamic diameter.

### 2.6 Fusion of lipids with high-expression membranes

Membrane/lipid fusion was carried out using a freeze/thaw/sonication procedure [52]. A 2.5% (*w*/*v*) dispersion of 1-palmitoyl-2-oleoyl-*sn*-glycero-3-phosphocholine (POPC) or *E. coli* total lipid extract (Avanti polar lipids) was prepared in 20 mM Tris (pH 8) by vortexing followed by incubation at 42 °C for 15 min. Each dispersion was then sonicated on ice with a probe sonicator for a total of two minutes, alternating 10 s on and 10 s off to prevent overheating. These lipids were mixed 5:1 with photosynthetic membranes containing RC-LH1-X complexes isolated from cells grown under high expression conditions. The membrane/lipid mix was vortexed and subjected to five rounds of freezing in liquid nitrogen and thawing for 15 min whilst sonicating in an ultrasonic bath at room temperature. Each sample was then sonicated with a probe sonicator for 2 min on ice, pulsing 10 s on and 10 s off, followed by five more rounds of freeze-thaw and one further round of probe sonication.

For fusion of DD13 membranes with RC-LH1-X high expression membranes, the two types of membrane were prepared to the same OD at 650 nm, mixed in a 1:1 ratio and diluted 2.5 fold in 20 mM Tris (pH 8). Samples were then subjected to the same procedure of a total of ten freeze-thaw cycles and two probe sonication cycles described above.

For preparative-scale fusion of fusion of DD13 membranes with RC-LH1-X high expression membranes, 50 mL of each were mixed. The solution was divided equally between three 50 mL Falcon tubes, frozen in liquid nitrogen for 5 min and placed in a bath sonicator for 45 min at room temperature. This freeze/thaw cycle was repeated five times. After the final thaw each solution was sonicated with a probe sonicator on ice for a total of 52 mins per tube, pulsing with cycles of 2 min on and 5 min off to prevent overheating. Five more rounds of freeze-thaw were performed followed by one more round of probe sonication. RC-LH1-X complexes were purified from these fused membranes using SMA as described above.

A volume of 75 μL of each sample was used to perform a SMA solubilisation assay with 2.5% (*w*/*v*) 10 kDa Xiran SZ30010.

### 2.7 Transmission electron microscopy

Copper grids for negative stain were prepared by the carbon flotation technique. Samples were diluted to ~ 0.05 mg/mL in 20 mM Tris (pH 8.0) and small aliquots were adsorbed on carbon-coated mica. The mica was then transferred to a staining solution containing 2% (*w*/*v*) sodium silico tungstate, causing detachment of the carbon film. Subsequently, a copper grid was placed on top of the detached carbon which was recovered and dried under air flow. Images were taken under low dose conditions at a nominal magnification of 23,000 × or 30,000 × with a T12 electron microscope (FEI, Hillsboro, OR) at an operating voltage of 120 kV using an ORIUS SC1000 camera (Gatan, Inc., Pleasanton, CA).

### 2.8 Thin layer chromatography and lipid analysis

Four independent lipid extractions were performed from the same batch of solubilised RC-LH1 after fusion with DD13 membranes, and from high expression membranes, by the Bligh and Dyer method [53]. Extracted lipids were deposited onto a silica TLC plate (MACHEREY NAGEL GmbH & co) using a Linomat 5 sample applicator (Camag). The TLC plate was developed using a solution of chloroform:methanol:acetic acid:water (85:15:10:3.05) in an ADC2 Automatic Development Chamber (Camag). Lipids were visualized by dipping the plate into a methanol solution of 10% copper(II) sulfate in 8% sulfuric acid (98%), and 8% phosphoric acid (85%) and then drying the plate by heating at 130 °C for 12 min. Relative intensities were determined by densitometry using Quantity One (BioRad).

## 3 Results

### 3.1 Efficiency of RC extraction by different formulations of SMA

Our previous report on RC isolation and purification employed SMA2000, a 2:1 styrene:maleic acid formulation with an average molecular weight of 7.5 kDa [8]. However, as outlined in Table 1, Table 2:1 and 3:1 SMA copolymers are commercially available in a range of weight average molecular weights from 7.5–120 kDa, and low molecular weight 1.5:1 and 4.5:1 formulations are also available. As described in Materials and Methods, all eight copolymers listed in Table 1 were tested for their abilities to solubilise His-tagged wild-type RC complexes from membranes prepared from a strain of *R*. *sphaeroides* that lacks the LH1 and LH2 light harvesting proteins. Each SMA polymer was used at a final concentration of 2.5% (*w*/*v*). This was the highest concentration practicable given that limited solubility prevented the preparation of stock solutions of the long-chain SMA variants (Xiran SZ26080 and Xiran SZ26120) at concentrations > 3.5 to 4% (*w*/*v*).

**Table 2:** Lipid analysis by thin layer chromatography.

lipids	high expression RC-LH1-X membranes	SMA solubilised RC-LH1-X material^a^
CL	5.5 ± 0.6^b^	11.0 ± 1.5
PE	34.4 ± 1.4	30.4 ± 1.9
PG	22.6 ± 0.4	21.2 ± 0.6
PC	28.2 ± 0.3	25.8 ± 2.0
SQDG	9.3 ± 1.2	11.6 ± 2.2

a Material from fused membranes formed from a mixture of high expression RC-LH1-X membranes and DD13 membranes, solubilised using SMA and separated by nickel affinity chromatography.

b Percentage lipid composition based on densitometry of band intensities in TLC analysis.

**Fig. 2:**
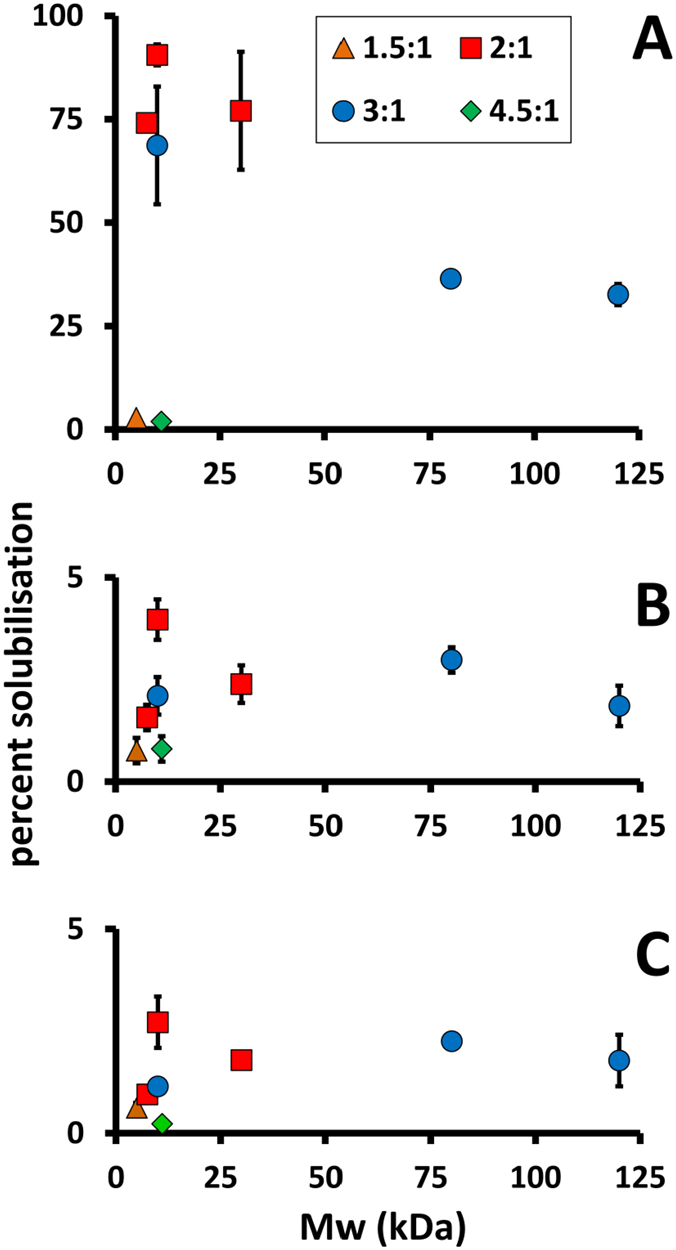
Efficiency of membrane protein solubilisation using SMAs of different average molecular weights. (A) RCs. (B) RC-LH1-X complexes. (C) RC-LH1 complexes. Symbols are used to denote different ratios of styrene to maleic acid; details of the eight polymers surveyed appear in Table 1. Error bars show standard deviations (three replicates).

Solubilisation efficiency was estimated by absorbance spectroscopy of the starting membrane material and the extracted soluble fraction, utilising the strong and distinctive spectroscopic properties of the RC bacteriochlorin cofactors to determine protein concentration. The soluble fraction was separated from unsolubilised membrane material by ultracentrifugation at 100,000 g for 2 h at 4 °C. All 2:1 or 3:1 variants of SMA with a molecular weight of 30 kDa or below were effective to similar extents, extracting 70–90% of RCs (upper left in Fig. 2A (and see Supplementary Table 1)). The two versions of the 3:1 formulation with much higher average molecular weights (80 and 120 kDa) were also able to solubilise RCs but at a significantly lower efficiency than the 10 kDa version (right in Fig. 2A). Low molecular weight SMAs with a 1.5:1 or 4.5:1 ratio of styrene to maleic acid were unable to solubilise RCs to a significant degree (bottom left in Fig. 2A). The inactivity of 1.5:1 SMA was consistent with recent reports that this formulation is unable to fully solubilise model membranes [21], [54]. The conclusion, therefore, was that low molecular weight 2:1 or 3:1 SMAs are the most effective for extraction of RCs, but longer chain variants with the same styrene to maleic acid ratio were able to achieve a somewhat less efficient extraction. These findings are consistent with those of Morrison et al. on SMA solubilisation of three *E. coli* membrane proteins [55].

### 3.2 Efficiency of RC-LH1-X complex extraction by different formulations of SMA

The same assay was carried out using intracytoplasmic membranes from a *R. sphaeroides* strain containing RC-LH1-X complexes but lacking LH2. As shown in Fig. 2B, for this protein the solubilisation efficiency was < 4% for all SMAs tested (and see Supplementary Table 1). One possible reason for this could be that the RC-LH1-X complex is too large to be accommodated in a SMA/lipid nanodisc, a monomer of the native RC-LH1-X complex being expected to have approximately three times the mass and twice the diameter of a RC at ~ 300 kDa and ~ 13 nm (Fig. 1). As outlined above, it has been reported that SMAs typically form discs of 10–15 nm diameter when incorporating proteins, and so a ~ 13 nm diameter monomeric RC-LH1-X complex may be too large to encapsulate. An additional factor could be that native RC-LH1-X complexes form dimers when in the native membrane [27], [30], [31], [35], [36], which doubles their mass and increases their diameter along the long axis to ~ 21 nm [27] (Fig. 1A, B).

To explore this latter point we also conducted a survey of SMA extraction efficiencies using membranes from an LH2-deficient strain containing RC-LH1 complexes lacking the PufX protein, which results in the assembly of exclusively monomeric RC-LH1 complexes in which the LH1 forms a complete ring around the central RC (Fig. 1E). The efficiency of extraction of this type of complex was also uniformly low (Fig. 2C (and see Supplementary Table 1)). This showed that the possibility that native RC-LH1-X complexes assemble in a dimeric arrangement in the membrane was not the reason for inefficient solubilisation by SMA. It should be noted that an increase in SMA concentration, or the length or temperature of incubation, had no effect on the low extraction efficiencies obtained for either RC-LH1-X or RC-LH1 complexes, nor did carrying out the extraction using membranes that also contained the LH2 antenna complex (data not shown).

### 3.3 Attempts to overcome the recalcitrance of RC-LH1-X membranes to solubilisation by SMA

In addition to their size, another factor that may prevent SMA solubilisation of RC-LH1-X complexes is an unfavourable membrane composition and/or organization. As oxygen is the primary regulator of photosynthesis gene expression in *Rhodobacter*, under semi-aerobic growth conditions the expression levels of the RC-LH1-X complex are high and protein crowding leads to the formation of highly-ordered RC-LH1-X arrays [31], [35], [36], [39], [40] (see schematic in Fig. 1F). Although high resolution structural information is not available for such membranes, it is likely that the amount of lipid bilayer in such protein-rich membranes is limited and there are extensive protein-protein interactions between adjacent RC-LH1-X complexes, producing a structure that has limited fluidity and limited opportunities for SMA to interact with contiguous regions of lipid bilayer.

**Fig. 3:**
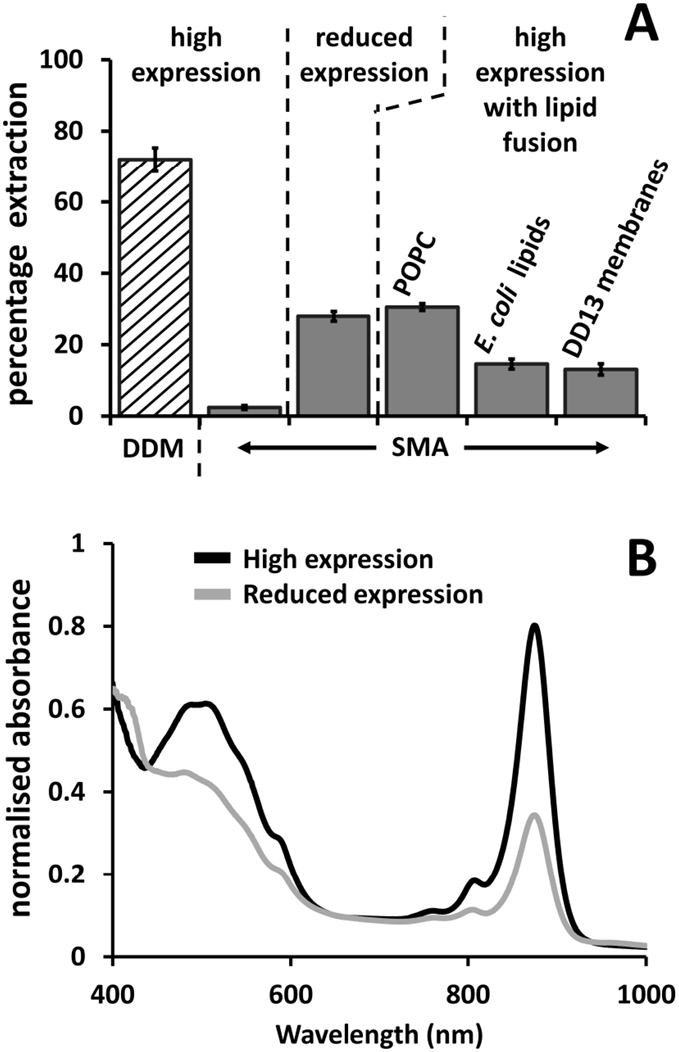
Efficiency of extraction of RC-LH1-X complexes. (A) Efficiency of solubilisation of RC-LH1-X complexes with DDM or the 10 kDa 2:1 SMA. Error bars show standard deviations (three replicates). (B) Absorbance spectra of membranes with high RC-LH1-X expression (black) and lowered expression achieved by growing cells at high aeration (grey). Spectra are normalised to the same membrane scatter at 650 nm.

It is known that RC-LH1-X complexes can be solubilised intact from highly-ordered LH2-deficient membranes by treatment with mild detergents such as *n*-dodecyl β-D-maltoside (DDM). To measure the maximal extent to which RC-LH1-X complexes can be extracted from the high-expression membranes prepared in the present work, these were treated with 2.5% (*w*/*v*) DDM. The mean extraction efficiency over multiple experiments was 72%, some 30-fold greater than that achieved with the same membranes using the 10 kDa, 2:1 formulation of SMA (Fig. 3A, high expression).

As the high degree of order shown by RC-LH1-X complexes is a consequence of their high concentration in the membrane [31], [35], [36], [39], [40], a possible way to enable more efficient extraction of RC-LH1-X complexes by SMA is to dilute their concentration by increasing the lipid-to-protein ratio. Experiments on membranes from a wild-type strain of *R. sphaeroides* fused with phosphatidylcholine (PC) liposomes have previously shown a loss of energy transfer from LH2 to LH1 when exogenous lipid is added, likely resulting from loss of protein-protein contacts and the formation of belts of lipids around individual complexes [56]. In the present work a number of methodologies were explored to achieve dilution of complexes in the membrane, the 10 kDa 2:1 formulation of SMA then being used to test the effect on solubilisation efficiency. In the first, cells of *R. sphaeroides* were grown at high aeration (see Methods) to down-regulate RC-LH1-X levels in the membrane (Fig. 3B). When complexes were extracted from these reduced expression membranes the solubilisation efficiency increased to 28%, some ~ 12-fold higher than was achieved with membranes prepared from high-expressing cells grown under standard semi-aerobic/dark conditions (Fig. 3A). In the second, high expression membranes were fused with pure 1-palmitoyl-2-oleoyl-*sn*-glycero-3-phosphocholine (POPC) lipid by subjecting a mixture of the two to multiple freeze/thaw/sonication cycles. This increased the solubilisation efficiency by ~ 13-fold over the same membranes without POPC treatment (Fig. 3A). Thus, it would appear that lowering the concentration of RC-LH1-X complexes in these membranes made them somewhat more amenable to solubilisation by SMA.

One attractive aspect of the use of SMA is the possibility of purifying proteins along with their immediate native lipid environment. Clearly, addition of a synthetic lipid such as POPC has the potential to disturb this environment and directly impact on native protein-lipid interactions that may be crucial for function or stability. Accordingly, the ability of two non-synthetic preparations to increase the efficiency of RC-LH1-X complex extraction was also tested. The first of these was a commercial preparation of total lipids from *E. coli*, which is predominantly phosphatidyl ethanolamine (PE) with lower percentages of cardiolipin (CL) and phosphatidyl glycerol (PG). Both *E. coli* and *R. sphaeroides* are gram-negative bacteria, and the latter also has PE as the main lipid with PG and CL, the main difference being that it also has phosphatidyl choline (PC) [8], [57], [58], [59]. This preparation of *E. coli* total lipid, when fused with high RC-LH1-X expression membranes by freeze/thaw/sonication, enabled an extraction efficiency of approximately 15% (Fig. 3A).

The next approach was to fuse high RC-LH1-X expression membranes with membranes purified from the DD13 strain of *R. sphaeroides* in which all photosynthetic complexes are absent. This DD13 strain has a deletion of the *puf* operon which abolishes RC and LH1 expression and of the *puc1BAC* operon which abolishes LH2 expression. This double deletion strain was used to make the RC and RC-LH1-X expressing strains used for the main part of the work described above (see Methods). This approach to dilution of RC-LH1-X high expression membranes enabled 13% extraction of RC-LH1-X complexes with SMA, similar to that achieved with *E. coli* total lipids (Fig. 3A). Although this efficiency was around half that achieved with pure POPC, the advantage was one of scale and cost, in that it was straightforward to produce a large quantity of DD13 membranes for fusion with high expression RC-LH1-X membranes, and therefore produce a large amount of SMA-solubilised RC-LH1-X complexes for further analysis.

Another advantage of this approach is that DD13 membranes comprised native *Rhodobacter* lipids and so should not markedly change the lipid environment of RC-LH1-X complexes during fusion and extraction. To verify this, thin layer chromatography (TLC) was carried out on lipid extracts of SMA solubilised material (see below) and lipid percentage compositions were estimated by densitometry. TLC of lipid extracts of complexes from high expression membranes (Table 2) identified PE, PC, CL, PG and sulphoquinovosyl diacylglycerol (SQDG), in accordance with the lipid profile of antenna-deficient membranes in *R. sphaeroides* [8]. The same five lipids were also detected in similar relative amounts in material from high expression RC-LH1-X membranes that had been fused with DD13 membranes, isolated using SMA and separated using nickel affinity chromatography, showing that the fusion process did not markedly change the lipid content of SMA-solubilised material. Only CL seemed to be slightly enriched in material solubilised from fused membranes, mostly at the expense of PE (Table 2).

We were also able to extract approximately 10% of RC-LH1-X complexes by fusing high expression membranes with a crude lipid extract from the same membranes (data not shown). However, mixing with this lipid extract caused some changes in the RC-LH1-X absorbance spectrum indicative of protein unfolding. This was likely due to the pigment-rich nature of RC-LH1-X membranes and the crude nature of this method of lipid purification (a 1:1 methanol:chloroform extract, which can also extract pigments and other hydrophobic components associated with the bilayer). Nevertheless these findings suggest that lipids chemically extracted from a recalcitrant membrane system could be used to make that membrane more amenable to solubilisation by SMA.

### 3.4 Characteristics of SMA-solubilised RC-LH1-X complexes

RC-LH1-X complexes solubilised using SMA from high-expression membranes, reduced expression membranes and from high-expression membranes fused with membranes from strain DD13, were separated from other solubilised membrane components by nickel affinity chromatography, making use of the His-tag on the RC M-subunit (see Methods). For comparison, RC-LH1-X complexes were also purified from high-expression membranes after solubilisation by DDM. Steady state and kinetic absorbance spectroscopy showed that membrane fusion/solubilisation had no significant impacts on the structural and functional integrity of the RC-LH1-X complex (data not shown).

**Fig. 4:**
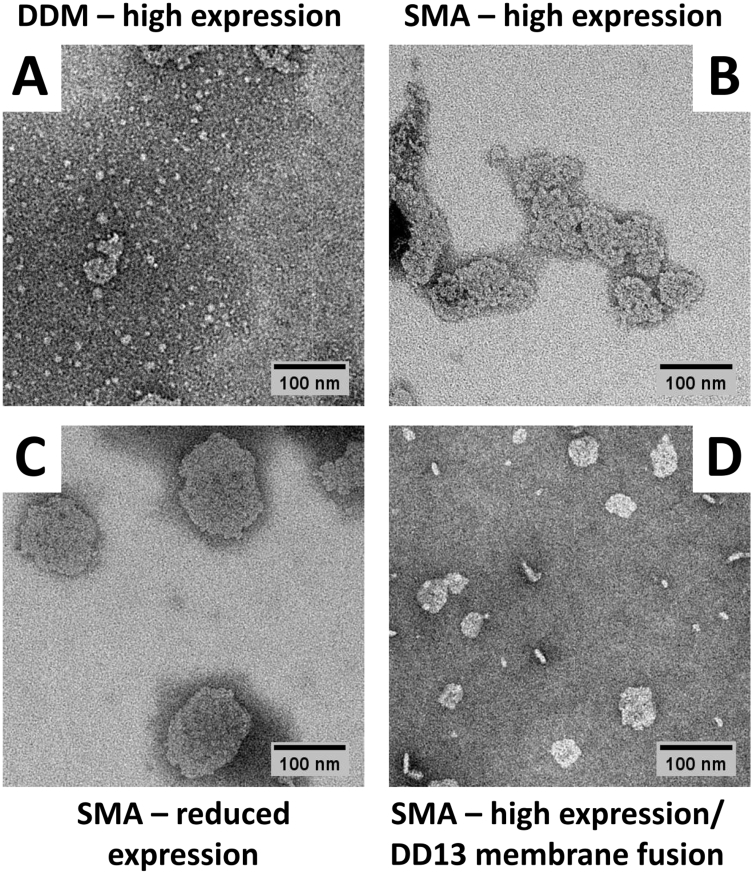
TEM images of RC-LH1-X complexes. (A) RC-LH1-X complexes purified in DDM detergent. (B) Material solubilised with SMA from untreated high-expression RC-LH1-X membranes. (C) Material solubilised with SMA from reduced expression RC-LH1-X membranes. (D) Material solubilised with SMA from high expression RC-LH1-X membranes that had been fused with DD13 membranes.

Transmission electron microscopy (TEM) of DDM-solubilised protein showed particles of dimensions consistent with monomeric isolated RC-LH1-X complexes (expected to be ~ 13 nm in diameter), a small portion of which had also aggregated into larger structures (Fig. 4A). It is likely that this aggregation was an artefact of the drying and staining procedures required for TEM as the parent solution had been prepared by size exclusion chromatography and did not contain aggregates. In contrast, TEM revealed that the SMA-solubilised material eluted from nickel affinity columns consisted mainly of small fragments of membrane rather than individual, discrete nanodiscs (Fig. 4B-D). Membrane fragments liberated in small amounts by SMA from untreated high-expression membranes were typically 50–100 nm in diameter, and some images showed internal, periodic structure consistent with the presence of densely-packed RC-LH1 complexes (Fig. 4B). Fragments solubilised in higher amounts from reduced expression membranes had similar characteristics (Fig. 4C). Strikingly, fragments isolated from high expression membranes that had been fused with DD13 membranes were markedly smaller, typically below 50 nm in diameter (Fig. 4D). The conclusion, therefore was that SMA-solubilisation of these highly ordered RC-LH1-X membranes resulted in the production of small membrane fragments rather than individual complexes in nanodiscs. These fragments were small enough to remain in solution during a standard clearing ultracentrifugation spin and the His-tagged RCs within were capable of interacting with the nickel affinity resin.

### 3.5 Extraction of synthetic RC oligomers with SMA

**Fig. 5:**
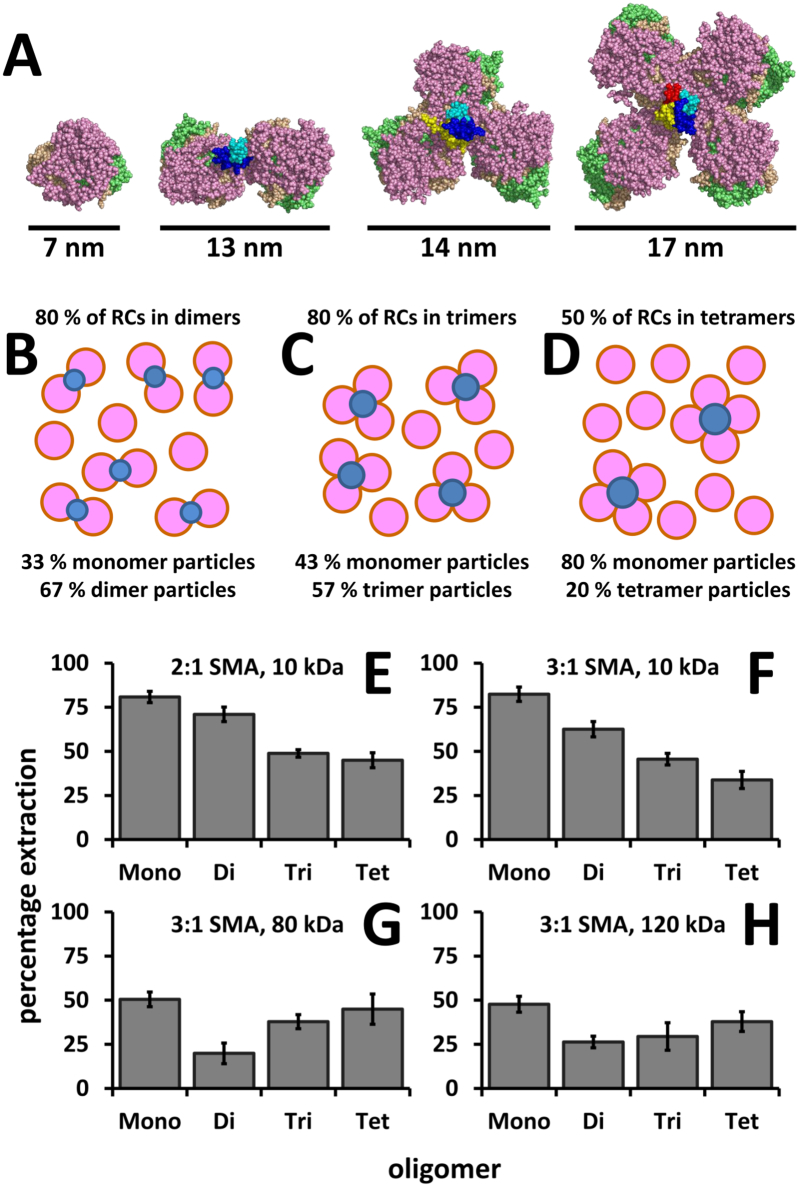
Efficiency of solubilisation of native monomeric and artificially oligomeric RCs with four variants of SMA. (A) Molecular models of monomeric, dimeric, trimeric and tetrameric RCs [44]. View is from the cytoplasmic side of the membrane, RCs are shown as in Fig. 1 with the fused coiled-coil bundle in blue/cyan for dimers, blue/cyan/yellow for trimers or blue/cyan/yellow/red for tetramers. (B) Relative populations of RC monomers/dimers. (C) Relative populations of RC monomers/trimers. (D) Relative populations of RC monomers/tetramers. (E) Extraction using 2:1 SMA, 10 kDa. (F) Extraction using 10 kDa 3:1 SMA. (G) Extraction using 80 kDa 3:1 SMA. (H) Extraction using 120 kDa 3:1 SMA. In panels (*E*-H) Mono refers to extraction from antenna-deficient membranes containing native monomeric RCs whilst Di, Tri and Tet refer to extraction from antenna-deficient membranes that contain engineered dimeric, trimeric and tetrameric RCs, respectively, and error bars show standard deviations (three replicates).

To explore further the issue of how size affects the efficacy with which SMA can isolate a membrane protein in the form of a nanodisc, a set of engineered RCs were used that assemble as synthetic, programmable dimeric, trimeric and tetrameric complexes. This was achieved as described in previous work by tethering monomeric RCs together through genetically-encoded fusion of the N-terminus of one of the component polypeptides to an extra-membrane α-helix that forms a water-soluble coiled-coil bundle [44]. The sequence of this α-helix determines the oligomeric state of the bundle [60], and hence that of the tethered RCs [44]. Together with the native monomer, these artificial oligomers provide a set of RC complexes with calculated masses of 104, 216, 324 and 436 kDa respectively, housed in an antenna-deficient membrane system that has been proven to be amenable to solubilisation with SMA2000 [8]. X-ray crystal structures of the oligomers have not been determined but molecular models of each of them in a bilayer have been constructed and validated by AFM images of individual oligomers [44]. Views of these molecular models perpendicular to the plane of the membrane are shown in Fig. 5A. In terms of gross dimensions in the plane of the membrane, monomeric and dimeric RCs can be represented by ellipsoids with approximate dimensions of 6 × 7 nm and 6 × 13 nm, respectively. Trimeric and tetrameric RCs can be represented by circles with diameters of 14 nm and 17 nm, respectively.

When isolated from antenna-deficient membranes using detergent, RCs modified in this way have been found to be a mixture of monomers and the programmed oligomer, with a yield of ~ 80% of the total RC population in the dimer or trimer form, and ~ 50% of the total RC population in the tetramer form (based on the separation of the monomeric and multimeric species by size-exclusion chromatography after enrichment of LDAO-solubilised proteins by nickel affinity chromatography) [44]. It should be noted that the percentage of RCs in the oligomeric form in membranes may be substantially higher than this as the extent to which detergent extraction causes monomerisation has not been determined. As illustrated in Fig. 5B-D, if a RC oligomer is regarded as a single macromolecule, these minimal estimates mean that “dimer membranes” contained 67% RC dimers and 33% RC monomers (Fig. 5B), “trimer membranes” contained 57% RC trimers and 43% RC monomers (Fig. 5C), and “tetramer membranes” contained 20% RC tetramers and 80% RC monomers (Fig. 5D).

As shown in Fig. 5E and F, lower molecular weight 2:1 and 3:1 SMA formulations that achieved > 80% extraction of RC monomers were also able to extract RCs from membranes containing a proportion of artificially oligomeric RCs, but the efficiency of extraction decreased as the oligomer became larger. Hence, organising a sub-population of RCs into larger structures in the membrane impacted on the ability of SMA to liberate the total RC population into solution. Such a gradual decline in extraction efficiency could not be accounted for simply by an inability of the SMA to solubilise RCs when in the oligomeric form, as this should be expected to cause a much sharper drop-off of efficiency for membranes containing dimers and trimers (where the monomer RC population corresponds to < 20% of the absorbance in the starting material), and a recovery to higher extraction efficiency for tetramers (where the monomer population is ~ 50% of the “total” absorbance). The conclusion therefore, is that although a decrease in the proportion of monomeric RC complexes may have contributed to the gradual reduction in extraction efficiency across the four types of membrane, it was likely that artificial tethering of RC complexes into larger and larger oligomers also contributed to this reduction, these formulations of SMA gradually becoming less effective at solubilising RCs as the size of the oligomer became larger.

Interestingly, a different trend was seen when the longer chain formulations of SMA were used (Fig. 5G and H). Here there was a sharp drop-off in extraction efficiency on moving from monomers to dimers, and the extraction efficiency seemed to recover somewhat with trimers and then tetramers. This trend may be related to the relative size of the monomer population, which changes through 100% to 20% to 20% to 50% on moving from monomers through to tetramers, but a clear correlation could not be drawn. However, it was possible to conclude that a simple correlation with oligomer size was no longer seen. Intriguingly, focussing just on the data for monomeric and tetrameric RCs in Fig. 5E-H, although the longer chain SMAs were clearly less effective at solubilising monomers than the shorter chain SMAs, the efficiency of extraction from membranes containing RC tetramers was similar for the long and short chain forms. When solubilised with DDM all RC-oligomer variants were extracted to a ~ 70% or greater efficiency (data not shown), again showing that these effects are specific to SMA. As the chain length of SMA is not thought to influence the particle size [21], it is likely that the intrinsic mechanisms of solubilisation for these polymers is responsible for this trend.

### 3.6 Characteristics of SMA-solubilised RC oligomers

Using a protocol previously reported for the purification of monomeric RCs in SMA [8], membranes expressing engineered dimeric, trimeric and tetrameric RCs were treated with 2:1 SMA (10 kDa from Polyscope) and the solubilised RCs were purified using nickel affinity chromatography. Due to the high mass of the SMA/lipid/protein nanoparticles it was not possible to separate nanoparticles containing multimers from those containing monomeric RCs in the way that is possible when RC monomers and oligomers are solubilised using LDAO.

DLS showed that nanoparticles prepared from membranes containing RC dimers were similar in size to those prepared from membrane containing exclusively RC monomers, with diameters of 12 ± 10 nm and 11 ± 8 nm, respectively. For the latter this value was similar to that of 12 ± 7 nm reported by us previously for RC monomers prepared with a similar, but not identical, formulation of 2:1 SMA (SMA2000, 7.5 kDa from Cray Valley) [8]. Also based on DLS, SMA/lipid nanoparticles purified from membranes containing RC trimers and tetramers were consistently somewhat larger at 14 ± 8 nm and 13 ± 16 nm, respectively. As indicated above, modelling suggests that RC trimers and tetramers can be approximated in the plane of the membrane by circles of the order of 14 and 17 nm diameter, respectively, which would explain the need for a somewhat larger nanoparticle than is required to accommodate RC monomers or dimers. It should be remembered that DLS measures an average hydrodynamic radius and so does not simply report on the diameter in the plane of the membrane. In addition, it is probable that each preparation of purified SMA/lipid/RC nanoparticles contained a substantial fraction of RC monomers in addition to RC oligomers, which could lead to an underestimate of the diameter of the sub-population of nanoparticles accommodating oligomers. This was particularly the case for tetramers as in this system the oligomeric form made up only 20% of the RC complexes in the membrane when viewing the RC tetramer as a single molecule.

TEM of these nanoparticle preparations showed a range of particle sizes (data not shown), in broad agreement with the findings from DLS. However, it was not possible to confirm from this imaging whether some of these particles housed oligomeric RCs.

## 4 Discussion

Systematic investigations of the mechanism by which SMA solubilises membranes have employed liposome systems comprised of pure lipids [61], [62], [63], [64], [65]. There it was found that the polymer inserts into the membrane after which the hydrophobic styrene groups intercalate with the lipid tails [61], [62], [66], [67]. The resulting nanodiscs have a mean diameter of approximately 9 nm and are narrowly distributed. Nanodiscs incorporating proteins tend to be somewhat larger, at 10–15 nm diameter with some reports of structures up to 24 nm. These data indicate that intrinsic curvature of SMA may be involved in the formation of nanodiscs when inserted into a lipid bilayer, but that the degree of this curvature is somewhat flexible to allow the incorporation of membrane proteins through the formation of larger discs than are seen in ideal lipid-only systems. In this report, we were able to use SMA to solubilise engineered oligomeric RCs that have an expected diameter of up to 17 nm and comprised up to 44 membrane-spanning α-helices. However, given the challenges we encountered in isolating RC-LH1-X and RC-LH1 complexes from their native membranes it remains to be seen whether they can be accommodated in a SMA nanodisc.

Examination of the literature shows that most studies have employed SMA formulations with a 3:1 or 2:1 ratio of styrene to maleic acid. In the present work, both types were equally effective in solubilising monomeric RCs when average chain molecular weights were below 30 kDa. However, neither a 1.5:1 nor a 4.5:1 formulation was able to solubilise monomeric RCs. A simple way to rationalise this would be to postulate that the 4.5:1 version is insufficiently hydrophilic to make efficient initial ionic interactions with the lipid headgroups, whilst the 1.5:1 version is insufficiently hydrophobic to insert into the membrane to the extent required to form the nanodisc. Therefore, it is likely that the ratio of hydrophobic to charged groups required to form a SMA nanodisc occupies a small window. Interestingly high molecular weight 3:1 SMAs (80 and 120 kDa) were less effective at solubilising monomeric RCs than their < 30 kDa counterparts, but this trend was lost when RCs were tethered together into synthetic tetramers. This suggests that there may be some advantage to using longer chain variants of SMA for the purification of larger complexes.

**Fig. 6:**
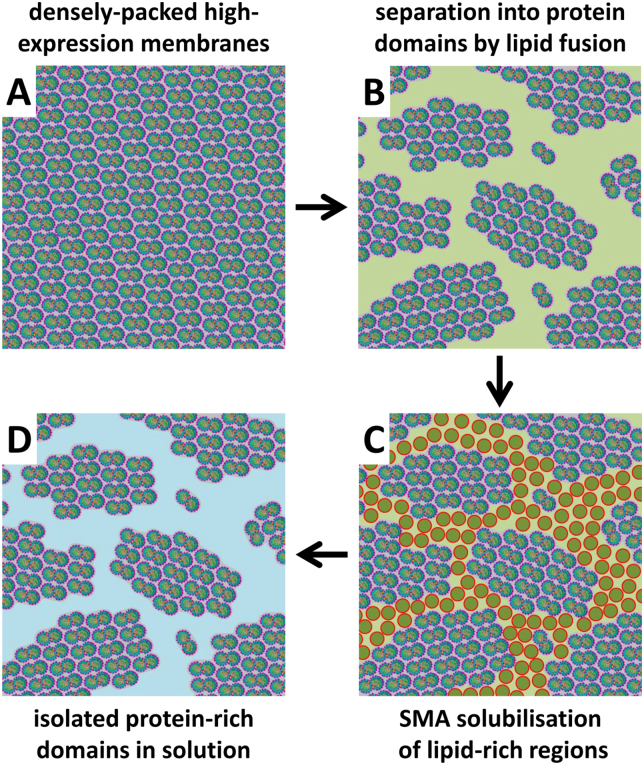
Model for formation of membrane patches on SMA treatment. (A) SMA-resistant high-expression RC-LH1-X membranes have a low lipid:protein ratio and limited regions of lipid bilayer. (B) Fusion with lipids or SMA-amenable bilayer-rich membranes introduces lipid-rich regions (pale green) between domains of closely packed RC-LH1-X complexes. (C) Addition a of SMA causes solubilisation of bilayer rich regions as SMA-lipid nanodiscs (red/olive green). (D) This treatment liberates protein-rich membrane fragments that are sufficiently small to stay in solution (blue) during clearing ultracentrifugation spins and pass through the matrices of chromatography columns.

As outlined above, initial failures to achieve substantial solubilisation of RC-LH1-X complexes from photosynthetic membranes using any of the SMA variants could be overcome by reducing the level of protein expression or by fusing high-expression membranes with pure lipids, lipid extracts or less SMA-recalcitrant membranes from the same organism. The likely explanation is that these treatments reduced the density of packing of RC-LH1 complexes in the membrane enabling permeation by SMA, which suggests that SMA needs regions of lipid bilayer in order to at least initiate nanodisc formation. However, in contrast with our previous findings with RCs extracted from antenna-deficient membranes, the RC-LH1 complexes solubilised by these treatments did not consist of a uniform population of individual proteins housed in nanodiscs but rather clusters of proteins housed in membrane fragments. These fragments were sufficiently small not to be sedimented by a standard membrane clearing spin (1 h at 150000 RCF) and to be able to pass through the matrix of a Ni-affinity chromatography column. As shown in Fig. 6, our hypothesis is that in these cases the fusion of densely-packed RC-LH1-X membranes (panel A) with lipids creates small islands of closely packed RC-LH1-X complexes separated by regions of bilayer (Fig. 6B), and solubilisation is achieved by SMA encapsulation of lipids in these bilayer regions (Fig. 6C), leaving membrane patches, possibly associated with some SMA polymer, free in solution (Fig. 6D). Given this, it may be possible to isolate a greater proportion of individual RC-LH1-X/SMA nanodiscs from SMA-recalcitrant membranes either by further increasing the lipid-to-protein ratio beyond that achieved in the present study or by applying additional treatments to induce more extensive mixing of lipid-rich and protein-rich sub-domains within such lipid-diluted membranes. Ordwyck-Rydmark and co-workers have shown that SMA can be used for the solubilisation of bacteriorhodopsin following fusion of purple membranes with DMPC [65], and in a recent study of the use of SMA for purification of bacteriorhodopsin expressed recombinantly in *Escherichia coli* it was shown that the yield of protein could be increased by fusing *E. coli* membranes with 1.5% (*w*/*v*) DMPC as a powder or as liposomes [68].

We conclude that successful solubilisation of a membrane protein by the SMA copolymer requires the right balance of copolymer hydrophobicity/hydrophilicity and length, and that properties of the target membrane such as lipid:protein ratio and the degree of order shown by component proteins are also of crucial importance. Our data also suggest that SMA is a “gentle” solubilising agent, which can preserve weak protein-protein interactions such as those involved in the formation of synthetic RC oligomers. This highlights the utility of SMA not only as a tool to isolate discrete individual complexes but also to preserve the larger scale architectures within membranes that are susceptible to disruption by traditional detergents. As an example, SMA has been used to establish that the native architecture of the LHCII light harvesting complex from spinach is trimeric, and investigate properties that are dependent on native protein-lipid interactions that are not preserved in detergent [69]. This said, it has recently been shown that SMA does not preserve supercomplexes formed between cytochrome *c* oxidase and the cytochrome *bc*_1_ complex, although it does enable purification of cytochrome *c* oxidase along with two weakly-bound proteins, Rcf1 and Rcf2, known to be important for supercomplex assembly [13]. Important factors for determining whether a protein-protein interaction is preserved or disrupted by SMA may be the extent to which these interactions extend into the membrane interior or the hydrophobic/hydrophilic interface regions, and the amount of lipid present at the protein-protein interface. That some protein-protein interactions cannot easily be disrupted by SMA was evidenced by the fact that we were unable to isolate significant amounts of RC-LH1 proteins from bilayer membranes known to contain densely-packed and highly-ordered protein complexes, and therefore by inference low proportions of lipid bilayer. As it presently stands, therefore, SMA is not a panacea for membrane protein solubilisation. Although the various strategies we have explored have helped somewhat in identifying strategies for isolating proteins from SMA recalcitrant membranes, future optimisation of the SMA technology will be needed to reliably enable purification of differently sized proteins from membranes with limited amounts of lipid bilayer, and to enable purification of proteins in a fully functional form, particularly if they are required to undergo significant conformational changes as part of their mechanism. In closing, it should be acknowledged that SMA recalcitrance may in fact also be useful as a first purification step, in the sense that it allows removal of SMA-soluble fractions. This approach proved to be useful for obtaining fractions enriched in Photosystem (PS) I-light-harvesting chlorophyll (LHC) II supercomplex from spinach [15] and may also prove to be a useful property for isolation of domains such as lipid rafts that, in contrast to surrounding, fluid, SMA-amenable regions of the bilayer, are resistant to disruption by this copolymer [70].

The following is the supplementary data related to this article.

Supplementary Table 1 Percentage extraction of complexes with various SMA preparations. Supplementary Table 1

## Transparency document

Transparency document. Image 1
