# Supplementary material for: The effectiveness of styrene-maleic acid (SMA) copolymers for solubilisation of integral membrane proteins from SMA-accessible and SMA-resistant membranes
Source: Biochim Biophys Acta. 2017 Oct;1859(10):2133–43. doi: 10.1016/j.bbamem.2017.07.011 (PMC5593810; doi:10.1016/j.bbamem.2017.07.011)
Supplement: Supplementary Table 1 — Percentage extraction of complexes with various SMA preparations. [file mmc1.docx]

**SUPPLEMENTARY INFORMATION**

**The effectiveness of styrene**—**maleic acid (SMA) copolymers for solubilisation of integral membrane proteins from SMA-accessible and SMA-resistant membranes**

David J. K. Swainsbury^a^, Stefan Scheidelaar^b^, Nicholas Foster^a^, Rienk van Grondelle^c^,

J. Antoinette Killian^b^, Michael R. Jones^a,*^

**Supplementary Table 1. Percentage extraction of complexes with various SMA preparations**

| **ratio of styrene: maleic acid** | **average molecular**  **weight (Da)** | **RC** | **RC-LH1-X** | **RC-LH1** |
| --- | --- | --- | --- | --- |
| 1.47 | 5000 | 2.8 ± 0.3 | 0.8 ± 0.3 | 0.6 ± 0.1 |
| 2.00 | 7500 | 74 ± 2 | 1.5 ± 0.5 | 1.0 ± 0.6 |
| 2.16 | 10000 | 90 ± 14 | 4.0 ± 0.5 | 2.7 ± 0.2 |
| 2.16 | 30000 | 77 ± 7 | 2.0 ± 0.5 | 1.8 ± 0.2 |
| 3.19 | 10000 | 69 ± 5 | 2.1 ± 0.3 | 1.1 ± 0.2 |
| 3.02 | 80000 | 36 ± 2 | 2.9 ± 0.8 | 2.2 ± 0.4 |
| 3.02 | 120000 | 32 ± 10 | 1.9 ± 0.5 | 1.8 ± 0.3 |
| 4.53 | 11000 | 1.9 ± 0.9 | 0.8 ± 0.5 | 0.2 ± 0.1 |
